# Supplementary material for: Self-emergence of robust solitons in a microcavity
Source: Nature. 2022 Aug 10;608(7922):303–9. doi: 10.1038/s41586-022-04957-x (PMC9365690; doi:10.1038/s41586-022-04957-x)
Supplement: Supplementary file 1 — Supplementary Sections 1–3, including Figs. 1–10 and References. [file 41586_2022_4957_MOESM1_ESM.pdf]

nature portfolio

https://doi.org/10.1038/s41586-022-04957-x

Supplementary information

Self-emergence of robust solitons in a microcavity

In the format provided by the authors and unedited

Self-Emergence of Robust Solitons in Micro-Cavity

Maxwell Rowley[1], Pierre-Henry Hanzard[1], Antonio Cutrona[1,2,], Hualong Bao[1], Sai T. Chu[3], Brent E. Little[4], Roberto Morandotti[5], David J. Moss[6], Gian-Luca Oppo[7], Juan Sebastian Totero Gongora[1,2], Marco Peccianti[1,2] and Alessia Pasquazi[1,2*]

[1] Emergent Photonics (Epic) Lab, Dept. of Physics and Astronomy, University of Sussex, Falmer, BN1 9QH, England, UK
[2] Emergent Photonics Research Centre, Dept. of Physics, Loughborough University, Loughborough, LE11 3TU, England, UK
[3] Department of Physics, City University of Hong Kong, Tat Chee Avenue, Hong Kong, China SAR
[4] State Key Laboratory of Transient Optics and Photonics, Xi'an Institute of Optics and Precision Mechanics, CAS, Xi'an, China
[5] INRS-EMT, 1650 Boulevard Lionel-Boulet, Varennes, J3X 1S2, Québec, Canada
[6] Optical Sciences Centre, Swinburne University of Technology, Hawthorn, VIC 3122, Victoria, Australia
[7] SUPA, Department of Physics, University of Strathclyde, Glasgow, G4 0NG, Scotland, UK
*a.pasquazi@sussex.ac.uk

In these Supplementary Materials, we explore the slow, energy-dependent nonlinearities of the system and explain how these are the critical phenomena that allow the system to start-up into the desired soliton regime in a spontaneous way. As shown in the main manuscript, we experimentally observe the natural formation and robust preservation of laser cavity-solitons in our system, which indicates that they behave as dominant attractors. Very interestingly, such behaviour occurs also for continuous waves (CW) and Turing patterns. Although it is typical for Turing patterns to emerge from noise, the capability of the system to switch between the two families of states (Soliton/Turing) suggests the presence of a different stabilisation mechanism between the two regimes, which we control with the EDFA pump power.

Section S1 presents new modelling that captures this physics and, centrally, the role of the slow nonlinearities. This requires a new approach since the system of equations for the laser cavity-solitons of Refs[1–3] does not contain the physics of the slow phenomena. Here, we introduce a greatly expanded theoretical framework by adding a thermal nonlinearity in the microcavity as well as the gain nonlinearity in the amplifier, as obtained through a standard Maxwell-Bloch derivation. We show that these two energy-dependent nonlinearities effectively change the system's start-up diagram, permitting the generation of soliton states from noise – which is another way of saying spontaneous generation. This type of emergence occurs in this new system over an extensive range of initial phase detunings, highlighting the strong independence of the start-up process from initial phase conditions, in agreement with what we observe in our experimental characterisations.

In Section S2, we experimentally study the balance between the slow nonlinearities in the microcavity and that of the fibre laser. Starting from the state diagrams in Fig. 4 of the main text, we extract the variation in refractive indices for both the microring and laser cavities within the operating regime. These measurements show that the refractive index variations of the two resonators are of the same sign and within the same order of magnitude, confirming that their nonlinearities are both focusing and within the same range.

Finally, we study the temporal response of the system when regaining a stable stationary state after a disruptive event. These measurements highlight the difference in recovery times between pattern-like states and soliton states. The pattern states lock on the blue-detuned slope of the microcavity resonances and recover within the timescale of action typical of the thermal effect in the microcavity, approximately hundreds of microseconds. Conversely, the soliton states, which are locked on the red-detuned slope of the microcavity resonances, recover over the characteristic timescale of the Erbium gain dynamics, which is several milliseconds long. In agreement with the theory that we present in Section S3, these measurements confirm that the slow microring nonlinearity dominates the system when it converges to a blue-detuned pattern. At the same time, the amplifier nonlinearity governs the regime leading to red-detuned soliton states.

S1. Modelling

The theoretical analysis of this paper starts from the mean-field model used in Refs[1-3], consisting of a coupled system of dissipative nonlinear equations[4-8], which we expand here by adding the description of the slow, energy-dependent nonlinearities.

The complex variable a represents the field in the microcavity. A lossy nonlinear Schrödinger equation models the evolution of this variable in the time and space coordinates t and x. The spatial coordinate x is normalised to the microcavity length, and the equation has periodic boundary conditions for $x \in [0,1]$. The temporal propagation coordinate is, for convenience, normalised to the fibre cavity roundtrip time. The field in the main amplifying loop is described as a superposition of *2N+1* supermodes $b_q$which are periodic with the microcavity length and are modelled with a set of lossy linear dispersive equations for every supermode b_q. In this way, both a and b_q are defined in terms of the microcavity normalised spatial coordinate $x \in [0,1]$. Neglecting the group velocity mismatch for simplicity, we have

$$\partial_t a = \frac{i\zeta_a}{2}\partial_{xx}a + 2\pi\, i\, \Delta_a a + i\,|a|^2 a\ - \kappa a + \sqrt{\kappa}\sum_{q=-N}^{N} b_q, \tag{1}$$

$$\partial_t b_q = \left(\frac{i\zeta_b}{2} + \sigma\right)\partial_{xx}b_q + 2\pi\, i\,(\Delta_b - q)b_q + g\, b_q\ - \sum_{p=-N}^{N} b_p + \sqrt{\kappa}a. \tag{2}$$

The parameters Δ_a and Δ_b represent the normalised frequency detuning of the two cavities, g is the normalised gain (from 0 to 1), while the group-velocity-dispersion coefficients are $\zeta_{a,b}$, and the loss dispersion coefficient is σ, with values of $\zeta_a = 1.25\ \times 10^{-4}$, $\zeta_b = 3.5\ \times 10^{-4}$, $\sigma = 1.5\ \times 10^{-4}$. The coupling coefficient κ represents the physical number of amplifier modes which fall within the microcavity resonance, with $\kappa = \pi$ representing one mode per line and $\kappa = 2\pi$ two modes per line. Details on the derivation and normalisation of the system are in Refs[1-3], with further information in the footnote*.

To this basic model, we now introduce the effect of the slow nonlinear variables, which are generally dependent on the energies

$$E_a = \int_{-\frac{1}{2}}^{\frac{1}{2}} |a|^2\, dx\,, \qquad E_b = \sum_{q=-N}^{N}\int_{-\frac{1}{2}}^{\frac{1}{2}} |b_q|^2 dx.$$

For the fibre amplifier, we describe the gain evolution using a standard laser rate equation derived from the Maxwell-Bloch model under the standard fast polarisation decay and long-scale approximations that enable using an energy-dependent relationship [7]. Under these assumptions, the dynamics of the saturable gain g are governed by the slow constant τ_g (normalised to the fibre cavity roundtrip T_b) and the pumping gain g_P as follows:

$$\tau_g\, \partial_t g = -(1 + \eta E_b)g + g_P, \tag{3}$$

where η is a normalised inverse of the saturation energy. The variable η is used here as a control parameter for the effective energy saturation within the gain cavity. In practice, this parameter is

* We denote the cavity roundtrip times and free-spectral ranges, for the microcavity (A) and amplifying cavity (B) respectively, with $T_{(a,b)} = F_{(a,b)}^{-1}$, $F_a \approx 50$ GHz and $F_b \approx 80$ MHz. The normalised dispersions used here are $\zeta_a = -\beta_a v_a T_b T_a^{-2} = 1.25\ \times 10^{-4}$, $\zeta_b = -\beta_b v_b T_b T_a^{-2} = 3.5\ \times 10^{-4}$, and $\sigma = (2\pi\, \Delta F_F T_a)^{-2} = 1.5\ \times 10^{-4}$. Here $\beta_a \approx -20\ \mathrm{ps^2 km^{-1}}$ and $\beta_b \approx -60\ \mathrm{ps^2 km^{-1}}$(see Ref[9]) are the second-order anomalous dispersions for the waveguide and the fibre, respectively, ΔF_F =650 GHz is the band-pass filter bandwidth, and $v_a \approx v_b \approx 2 \times 10^8\ \mathrm{m\,s^{-1}}$ are the group velocities. In the Methods, to numerically fit the spectral soliton shapes we have used a sixth order dispersion to reproduce our flat top filter, with $\sigma_6 = (2\pi\, \Delta F_F T_a)^{-6} = (1.5\ \times 10^{-4})^3$. Note that the outpt port, as indicated in the Methods section, with the full system Eqs (1-2) reads $c(t) = \sum_{q=-N}^{N} b_q(t) - \sqrt{\kappa}\, a(t)$.

affected experimentally by any energy imbalance between the microcavity and the fibre cavity, which can be directly impacted by the main cavity losses. The variable g_P is directly determined by the optical pump: in practice, any variation in the parameter g_P accounts for experimental variations in the optical pump power.

The general evolution of the refractive index is taken into account by setting the two detuning variables in Eqs. (1,2) as

$$\Delta_a = \Delta_{Ta}, \qquad \Delta_b = \Delta + \Delta_{Tb} + \Theta\, g.$$

Here the variable Δ is a quantity corresponding to the initial relative detuning. The dependence on the gain occurs through the variable Θ, which is the atomic polarisation detuning scaled by the polarisation time decay T_P. As described, e.g., in Refs.[10,11], this quantity is obtained from the standard Maxwell-Bloch equations as $\Theta = (f_1 - f_0)T_P$, i.e., as the scaled detuning between the carrier frequency f_1 (in our experiments approximately the centre of the soliton, $\lambda_1 = cf_1^{-1}$ ~1545 nm, where c is the speed of light) and the atomic resonance frequency f_0 ($\lambda_0 = cf_0^{-1}$ ~1530 nm for Erbium). For an Erbium dopant, T_P varies in the range of 100 fs to 10 ps. In our experimental conditions, then, Θ is within the range $|\Theta| = [0.2, 20]$. Regarding the sign, we note that the refractive index decreases for wavelengths longer than the atomic resonance, implying a negative sign in our experimental conditions.

Following standard approaches[1,12–18], we model the thermal dependence of a cavity through a first-order equation with a direct dependence on the intracavity energy,

$$\tau_T \partial_t \Delta_{Ta} = -\Delta_{Ta} + \Gamma_T\, E_a. \tag{4}$$

We consider a focusing thermal nonlinearity with a positive sign. Here Γ_T is an effective nonlinear coefficient normalised to the microcavity Kerr nonlinear coefficient, and τ_T is a thermal time-decay constant. For the thermal nonlinearity in the fibre Δ_{Tb}, we can use a relationship analogous to Eq. (4). In practical terms, the relevant quantity is the detuning between the two cavities, namely

$$\Delta_b - \Delta_a = \Delta + \Theta\, g + \Delta_{Tb} - \Delta_{Ta}. \tag{5}$$

From Eq. (5), we see that a focusing thermal effect with a positive coefficient in the amplifier counteracts the (also) positive, focusing thermal effect in the microcavity (due to the minus sign in front of Δ_{Ta}). To simplify the analysis, and because the ratio between energies E_a and E_b is approximately constant in the stationary regime[1–3], we can compact the thermal modelling into a single first-order equation by imposing $\Delta_{Tb} = 0$. The physical, thermal coefficient of the microcavity is about two orders of magnitude larger than the Kerr nonlinearity. The cumulative Γ_T is, however, primarily compensated by the thermal nonlinearity in the amplifier. On this point, see the experimental analysis of Section S2.1: we detect the presence of a thermal nonlinearity in the amplifiers, which largely compensates for the thermal nonlinearity of the microcavity. Hence, we expect that a physically relevant value for the cumulative Γ_T will be within the range of a few times the Kerr nonlinearity.

For the thermal time decay constant, we use $\tau_T \approx 8 \times 10^3$, which in our experiments corresponds to the dominant timescale ~0.1 ms for the microcavity normalised to the roundtrip frequency of 80 MHz. The gain decay constant for Erbium[7] is ~ 10 ms resulting in $\tau_g \approx 8 \times 10^5$. In our numerical propagations, we set this constant to $\tau_g \approx 4 \times 10^4$, which is four orders of magnitude larger than the main cavity roundtrip time and is sufficient to observe the same kind of dynamics seen in the experiments.

S1.1 Effect of nonlocal nonlinearities on the soliton stability map: start-up strategies

The inclusion of Eqs. (3,4) does not change the nature of the stationary states of Eqs. (1,2). The latter can be readily recovered by using the steady-state detunings $\Delta_{Ta}(t \to \infty) = \Gamma_T E_a$ and gain $g(t \to \infty) = (1 + \eta E_b)^{-1} g_P$. In particular, if we consider a steady-state solution for Eqs. (1,2)

given the detuning $\Delta_a = 0, \Delta_b = \Delta_0$ and gain g_0, the same solution can also be used for Eqs. (1-4) if the pumping gain

$$g_P = (1 + \eta E_b)g_0 \tag{6}$$

is used, and the initial detuning is set to the value

$$\Delta = \Delta_0 - \Theta\, g_P + \frac{\Gamma_T}{2\pi} E_a. \tag{7}$$

Note that such a remapping preserves the stationary states but not necessarily their stability properties. We have verified numerically that stable states are preserved for the set of parameters employed here, with the same techniques used in Refs[1,2].

Similar to Fig. 2a in the main text, Fig. S1a shows the soliton stability region (in orange) as a function of the parameters Δ_0 and g_0, obtained from Eqs. (1,2) *without including the slow variables.* In this paragraph, we use $\kappa = 1.25\,\pi$, which models the experimental settings of Figs.1-3. The zero-energy state is a (trivial) solution of the system, and its instability regions are marked in blue (unstable) and white (stable), respectively. As discussed in the text, the zero-state instability region is particularly significant as it defines the set of parameters where the start-up is allowed. Stable, single solitons fall outside this region. Strictly speaking, this is mainly due to the tails of the temporal pulses sharing the instability properties of the zero state.

To understand the effect of Eqs. (3,4) on the system, the most straightforward method is to numerically integrate all the dynamical Eqs. (1-4) and observe the evolution of the parameters g and $\Delta_b - \Delta_a$ given by Eqs. (3,5). A typical trajectory for a system evolving into a stable soliton state is shown in Fig. 2a of the main text, which we will discuss at the end of this section. However, an alternative, more effective approach to the problem is to look at stability regions of the stationary states for the *whole system* of Eqs. (1-4) against the new, global variables of the system, i.e., the pumping gain g_P and the initial cavity detuning Δ.

Let us start from the trivial state. Since the energy of this state is zero, its stability region is directly remapped into $g_P = g_0$, and $\Delta = \Delta_0 - \Theta\, g_0$. The thermal Γ_T and gain saturation η variables do not play any role in such a remapping. Conversely, these variables directly affect the solitons, as discussed previously for Eqs. (6,7). Those relationships imply that we can modify the stability region boundaries and move them within the unstable region of the zero-state. It is important to note that, for Eqs. (1-4), the zero state no longer shares the stability properties with the soliton tails. Here we have separated the instability of the zero state at the start-up and at regime, where the background is again strictly stable, as needed for stable solitons. Such a situation is very different to the start-up approaches based on affecting the modulational instability spectrum of the trivial state, which allows only soliton crystals to be formed[1,19,20]. In that case, the background always remains modulationally unstable; hence, only a periodic solution can exist, as we discussed in Ref[5].

Figure S1b shows such a remapping for the typical parameters of our experiments. For the thermal detuning, we choose a positive coefficient, here $\Gamma_T = 5$, implying that the thermal nonlinearity of the microcavity dominates the system. This effect is usually detrimental to soliton formation, as it pushes the soliton region further into the stability region of the zero-state. However, we show that gain dynamics can compensate for it, thanks to two fundamental mechanisms. First, at high gain, it projects the stable soliton state into the unstable region of the zero-state, enhancing the possibility of a spontaneous start-up. Second, it pushes the soliton states into the red detuning region for a negative value of the coefficient Θ. Figure S1b shows such effect on the soliton stability region, where we used $\eta = 0.4$ and $\Theta = -13$ (as well as $\Gamma_T = 5$), which are within our experimental range (see footnote**). Eventually, the gain dynamics enables

** For this set of parameters, single soliton states have normalised energy $E_b \approx 0.9$, with slight variations across the stable area Hence, a value $\eta = 0.4$ allows saturating the gain with around one to two solitons, as we observe experimentally.

controlling the magnitude of the nonlinear refractive index *by acting on the pump gain.* In Fig. S1, the orange-filled area represents the stability region of the single soliton state. Since the remapping is energy-dependent, the stability regions vary with the number of solitons. The stability boundaries for two and three solitons are shown in dark blue and green lines, respectively.

As we noted above, in practice, the effective energy saturation coefficient η depends on the system losses. The parameter Θ is also affected by the spectral position of the bandwidth filter. Therefore, the gain evolution provides a set of independent control parameters that allow establishing a condition where the desired single soliton state mainly covers the start-up region of the system.

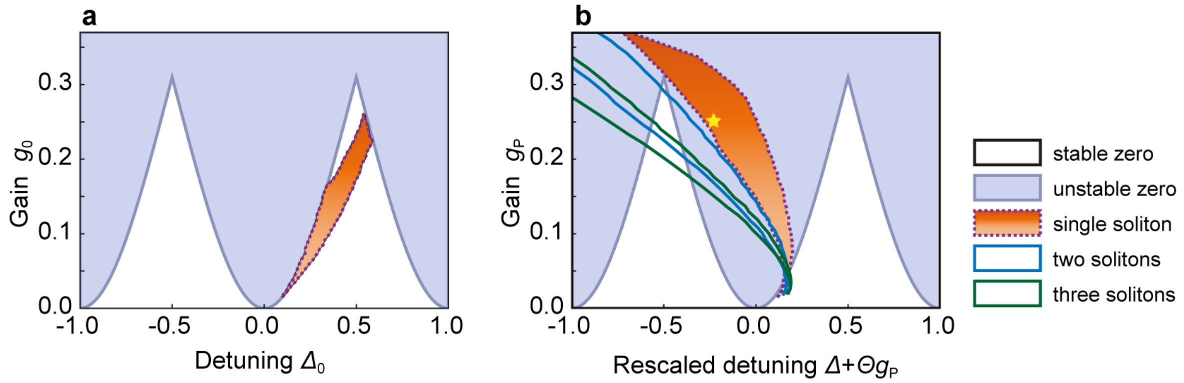

Figure S1 Stability region of the solitons and the zero state. The instability region of the zero-state is shown in blue and corresponds to the start-up region of the system. The stable regions for the zero and single soliton states are shown in white and orange, and a violet dashed line denotes the single soliton stability boundary. The dark blue and green lines indicate the stability region boundaries for the two and three soliton cases. Note that the stability regions of the solitons are periodical with Δ integer. Here, for simplicity, we depict the stability regions only in the baseband. **a** Diagram for Eqs. (1,2) without any slow nonlinearity or gain saturation. **b** Diagram for the full system, Eqs. (1-4). Here we use a predominantly focusing thermal nonlinearity in the microcavity and gain dynamics ($\Gamma_T = 5, \eta = 0.4, \Theta = -13$). The x axis is shifted by a factor $\Theta\ g_P$ for better visualisation. The yellow star indicates the coordinates used in the simulation of Fig. S2.

These considerations confirm that the solitons, including single soliton states, are compatible with an unstable zero state and can occupy a large part of the start-up region, accounting for the phase independence of the start-up that we observe experimentally. This configuration is a fundamental prerequisite for spontaneous start-up and is the most important result of this analysis. However, there may be other states that are allowed to start for a given set of parameters. These states may compete with the soliton state and eventually overcome it. The soliton's most obvious competitors are the CW solutions, but pulsed oscillations (e.g., relaxation oscillations) may also appear[15]. Besides, solutions with a different number of solitons may compete as well. The type of solution eventually produced by the system is the *dominant attractor* of the system for a given set of parameters. The competition among different states will also depend on the time constants τ_T, τ_g. A final answer to these questions requires a detailed stability study that will be the subject of a future theoretical work. Here, we restrict the discussion to the case of Fig. S1b, which summarises the main dynamics of our experiment, where we tested the behaviour of the system for g_P = 0.25. As predicted by the remapping of Fig. S1b, here, all cases initiating with a detuning falling within the single soliton range (here $\Delta + \Theta g_P \in [-0.28, -0.05]$) converge to a single soliton solution. Figure S2 shows the propagation of a typical solution, which corresponds to the attractor shown in Fig. 2a of the main text, where we converted the units of the detuning axis to the experimental units.

Figure S2a shows the spatio-temporal propagation of $|a|^2$, which is the intensity of the optical field in the microcavity. Figure S2b shows the temporal evolution of the peak intensity, while Figs. S2c and d depict the total detuning $\Delta_b - \Delta_a$, given by Eq. (5) and the gain g, given by Eq. (3), respectively. In the numerical integration, we superimposed a small white noise for the whole propagation to verify the robustness of the soliton. The attractor resulting from the latter two

quantities is depicted by the two-dimensional graph in Fig. S2e, in the variables $\Delta_b - \Delta_a$ and g. We plot the progressing timescales with colours changing from blue to yellow and superimpose it on the soliton and the zero-state stability region for the *local parameters* $\Delta_b - \Delta_a$ and g (which corresponds to Fig. S1a).

We observe that the system starts up purely from noise. The detuning moves towards the soliton region (positive values, which corresponds here to a red-detuning) while the energy in the system builds up, passing through a chaotic stage and forming three solitons. For this set of parameters, however, three solitons are not admitted as stationary states. Hence, the laser oscillation is lost. While dropping energy, the system falls again into the region where the zero background is unstable and, therefore, it can start up again. This cycle appears several times until the system enters the stability region with a single soliton, which is allowed and stably formed.

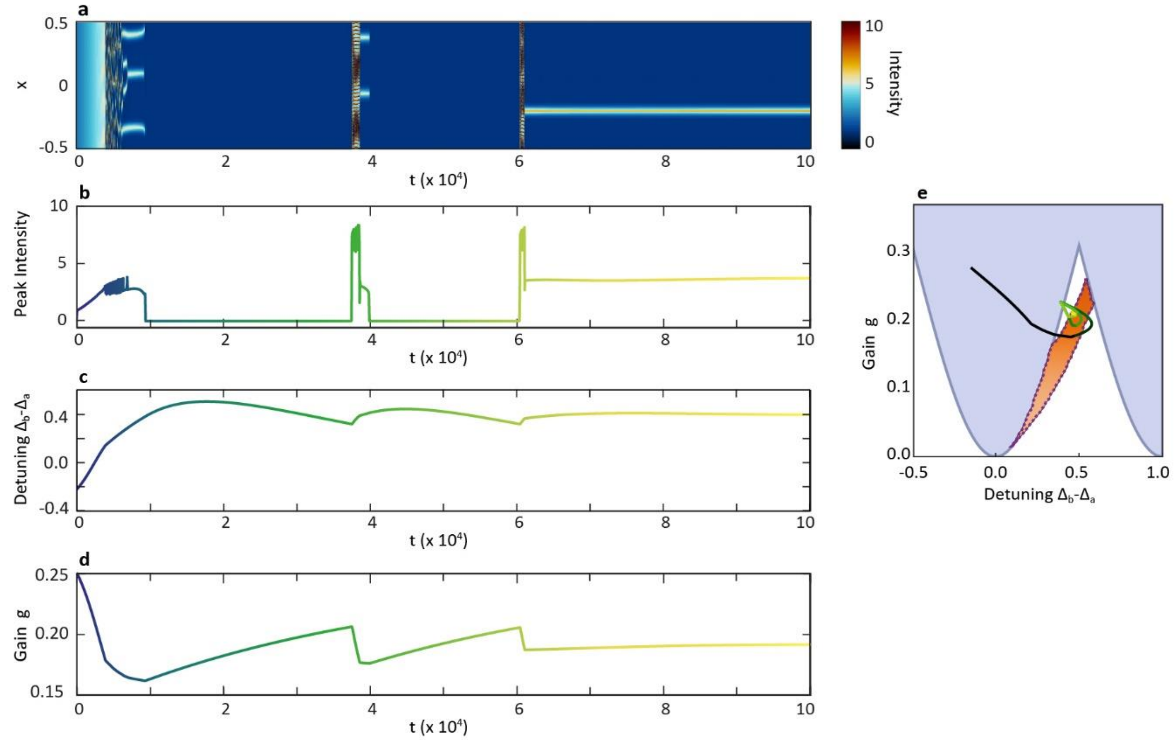

Figure S2 Start-up in the presence of gain-induced nonlinearity and thermal nonlinearity: single soliton formation. Numerical propagation for Eqs. (1-4), modelling a microresonator-filtered fibre laser with the inclusion of a saturable gain and gain-induced nonlinearity in the amplifying cavity and a thermal nonlinearity in the microcavity. The system parameters are $\Gamma_T = 5, \tau_T = 8 \times 10^3, \eta = 0.4, \tau_g = 4 \times 10^4, \Theta = -13, g = 0.25,$ and $\Delta + \Theta g_P = -0.21$. **a** Pseudo-colour map of the electric field intensity in the microcavity, in the normalised units of Eqs. (1-4), as a function of the position in the microresonator x (which is normalised against the microcavity roundtrips) and time t (which is normalised against the main-cavity roundtrips). **b** Temporal evolution of the peak intensity. The colours varying for increasing times matches with the plot inside panel e (showing the attractor). **c** Temporal evolution of the effective detuning $\Delta_b - \Delta_a$. The colours varying for increasing times matches with the plot in panel e. **d** Temporal evolution of the gain. The colours varying for increasing times matches with the plot in panel e. **e** Map of the attractor for the peak intensity, detuning and gain, as in panel c-d, following the colour code of panels b-d as a function of time. The attractor is superimposed to the soliton (orange) and zero state (blue) stability regions. Figure. 2a of the main text reproduces this map.

S2. Experimental investigation for variations in the global parameters

This section presents a detailed experimental investigation of the types of attractors in the system and their dependence on a set of the system's *global parameters* (laser cavity length, EDFA pump power, losses). Extending the results of Fig. 4 in the main text, we experimentally map the microcomb states against EDFA pump power and main cavity length while maintaining an environmental temperature variation of ±1 °C around the microcavity photonic chip and a fixed intracavity loss in the laser cavity. Specifically, we repeated the set of measurements four times in Fig. 4 e,f, (Section S2.1), keeping the intracavity losses at 16 dB, and then performed a second set at lower intracavity losses of 14 dB (Section S2.2).

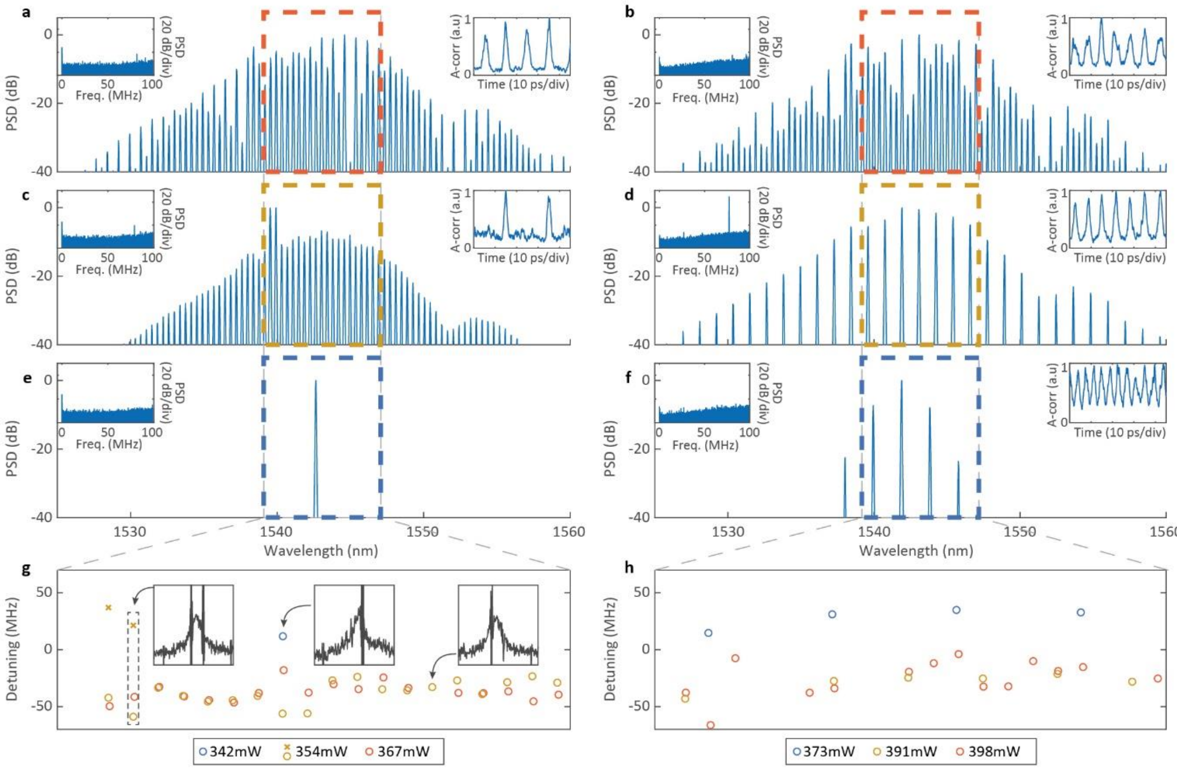

Figure S3 Summary of laser scanning-spectroscopy measurements. **a** Power spectral density (PSD) of optical and radio-frequency spectra (left inset) with autocorrelation (right inset) for an EDFA pump power of 367 mW, for the system with 16 dB losses. **b** Same as panel a, for an EDFA pump power of 398 mW, for the system with 14 dB losses. **c** Same as panel a, for an EDFA pump power of 354 mW. **d** Same as panel b for an EDFA pump power of 391 mW. **e** Same as panel a, for an EDFA power of 342 mW. **f** Same as panel b for an EDFA power of 373 mW. **g** The insets show three typical laser scanning spectroscopy measurements for a red-detuned oscillating line (right), blue-detuned line (centre), and coexistence of two oscillating modes (left). From these measurements, it is possible to extract the absolute frequency position of the oscillating line and the centre of the microcavity resonance. We define their difference as the frequency detuning of the lasing states in operating conditions. The distribution of the individual mode detunings across the wavelength span highlighted by the grey dashed lines for 16 dB intracavity losses and EDFA pump powers of 342 mW, 354 mW and 367 mW are shown in blue, yellow, and red, respectively. **h** Same as panel g, for the system at 14 dB intracavity losses, and for EDFA pump powers of 373 mW, 391 mW and 398 mW.

Figure S3 summarises the type of stable states that we observed for these two values of intracavity losses, with the cases on the left corresponding to the high loss condition (16 dB) and the case on the right corresponding to the low losses condition (14 dB). The general observation in these two sets is that, by reducing the losses, it is possible to reach a higher nonlinear regime. For the case of 16 dB, we observe CW states, as well as single and two soliton states, illustrated in Fig. S3a,c,e by the optical spectra together with the radio-frequency (RF) spectra (left inset) and autocorrelation (right inset). The RF spectra demonstrate excellent stability, and the autocorrelation shows a coherent, low background solution. For a loss of 14 dB, the typical, blue-detuned states that we observed featured Turing patterns (Fig. S3f), which appear from the modulational instability of a CW state and, hence, corresponds to a higher nonlinear regime

compared to the CW states of the case at 14 dB losses. When we further increased the pump energy, we observed red-detuned states, here three soliton states (Fig. S3b,d).

Figure S3g,h show three examples of laser scanning spectroscopy[1,21] measurements within the microcavity resonances corresponding to the examples in Fig. S3a-f. Direct experimental measurements of the laser scanning spectroscopy depicting the lasing modes within the microcavity are shown as insets in Fig. S3g. The right inset represents a red-detuned state, typically found in the spectral lines of the solitons, while the central inset shows a blue-detuned state, typically found in the spectral lines of the CW and Turing pattern cases. The left inset shows a typical case of coexisting blue and red lasing modes, which were observed in some soliton states.

Starting from a low Erbium-doped fibre amplifier (EDFA) pump power, in both Fig. S3g and Fig. S3h, we first find blue-detuned states corresponding to CW and Turing patterns. Note that the blue-detuned cases of Fig. S3h generally have more power and show larger blue-detuning values than those in Fig. S3g.

The soliton states have red-detuned oscillating lines. High energy soliton states, appearing at high EDFA pump powers, are entirely red-detuned. Interestingly, some single soliton cases show the coexistence with a few blue-detuned modes close to 1540 nm. This coexistence occurs in a spectral region where the system's gain has a strong and narrow peak. There, we observe a local maximum of the amplification due to the combined effect of the gain shape and intracavity spectral filtering. In this region, the EDFA exhibits a substantial dispersion of the nonlinear refractive index, which also decreases. We attribute to such two effects the presence of those modes superimposed to the red-detuned soliton modes.

We can now look at the complete experimental state diagrams of the system stationary states. We collected the measurements once the system stabilised after increasing the EDFA pump power for a fixed cavity delay. The *stationary state diagram* produced by compiling these measurements describes *the system's final state, naturally reached for* each parameter setting. Hence, this approach differs from typical measurements of transient states obtained during the sweeping of system's variables, such as the driving laser frequency detuning in externally driven configurations[22,23] or the gain current in self-injection locking[19].

We will first look in detail at the case of high losses (16 dB) in Section 2.1. There, we will also present a detailed study of the microcomb laser line frequency positions, highlighting some of the system's critical nonlinear properties. These include the measurement, under operating conditions, of the refractive indices for both the microring and laser cavitiy. These measurements show that the nonlinearities in the two cavities are of the same sign and order of magnitude. This is an important indication that the two nonlinearities are close to a balance point.

In Section S2.2, we present the stationary state diagram for the low losses (14 dB) case, which shows blue-detuned Turing patterns and red-detuned solitons. Within this range of parameters, we study the temporal response of the system when regaining a stable stationary state after a perturbation. These measurements highlight the difference in recovery times between the blue-detuned, pattern-like states and the red-detuned soliton states. The recovery times of the blue detuned states are compatible with the time of action of the thermal nonlinearity in the microcavity, confirming that this is the dominant effect in the system. The recovery time of the red-detuned solitons, conversely, is compatible with the response time of the Erbium gain in the amplifier, confirming that the gain-induced nonlinearity is dominating this regime.

S2.1 Experimental diagram of states at 16 dB losses: analysis of the laser scanning spectroscopy measurements.

Figure S4a shows a typical map highlighting the existence regions of the different states, here for intracavity losses of 16 dB. We identify two distinct zones that permit stable states – either continuous wave (CW) (blue) or broadband, soliton states (red to yellow region), appearing at low and high EDFA pump powers, respectively. In Fig. S4b, we report the optical spectra for all EDFA pump powers at a single relative cavity length setting (as indicated by the grey line in Fig. S4a). Specifically, we mark with a black dot the frequency position relative to the maximum value of each comb mode, and we report the resulting plot in Fig. S4b. Figure S4c reports three further repetitions of the same map, which show an impressive consistency. The map highlighted by the red background is labelled 'State diagram 2' and will be used in the following analysis to demonstrate the repeatability of the states obtained in our laser system.

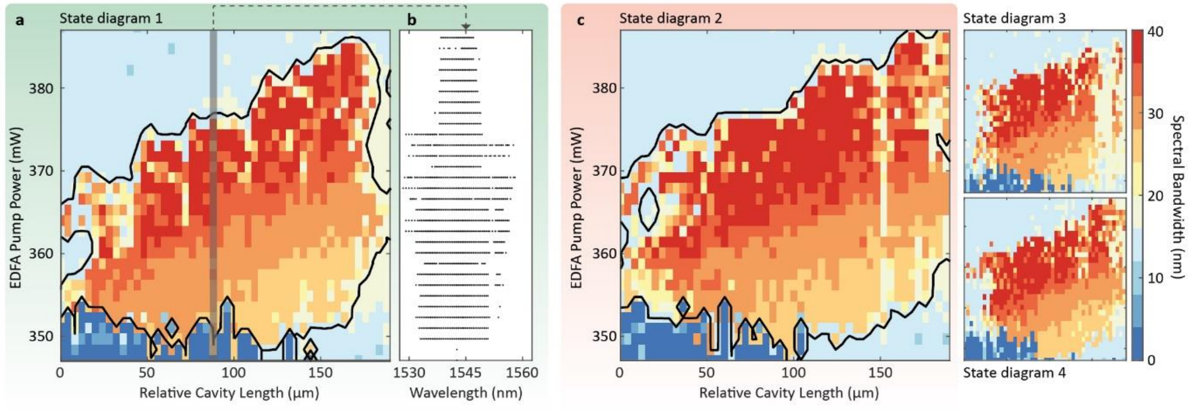

Figure S4. Repetitions of experimental state diagrams for the microresonator-filtered fibre laser with intracavity losses set to 16 dB. a Optical spectral bandwidth (calculated as the bandwidth at -40dB from the maximum) of the laser states, as a function of the cavity length and EDFA pump power. The soliton states are found in the yellow to red region with the broadest bandwidths, while CW states are in blue. Outside these regions, roughly defined by the black lines, the lasing regime is unstable. During the 10-hour experiment performed to acquire the data, the temperature was maintained at 40° C to within a fluctuation of a few degrees. **b** Optical spectra of the states along with the 92 μm delay position, identified by the grey line in panel a. The black dots represent the frequency position relative to the maximum value of each comb mode. **c** Repetition of the same experimental map, with two further repetitions on the insets, acquired immediately after the map in panel a, over subsequent 10-hour long experiments.

In Fig. S5, we show a set of additional measurements to supplement the maps in Fig. S4. Figure S5 depicts the gain (a,b) and EDFA output power (c,d), respectively.

We directly calculate the EDFA gain by measuring the intracavity losses and the microcavity output power (Fig. S5a,b). The latter generally increases as a function of the EDFA pump power before saturating in the upper region of the map when the system is outside the soliton region. There is also a discrete step in the EDFA gain level noticeable at the lower limit of the soliton region, corresponding to the boundary into the stable CW region.

We observe that the amplifier's output power or, equivalently, the input power to the microresonator, is roughly increasing with the EDFA pump power and has a negligible dependence on the free-space delay. It is also interesting to note that larger intracavity energies do not necessarily provide a state with a broader spectrum: the unstable states at the top of the map, as evident from Fig. S4, have a narrower bandwidth than the solitons, which appear to be the solutions with the broadest spectrum that the system can provide.

From the laser scanning spectroscopy measurements, we extract information about the refractive index of the two cavities. The quantities specifically shown here are the average frequency position of the comb mode around 1543 nm (Fig. S6a, b), the distribution of the average detunings (Fig. S6c, d), and the change in free-spectral range (Fig. S6e ,f).

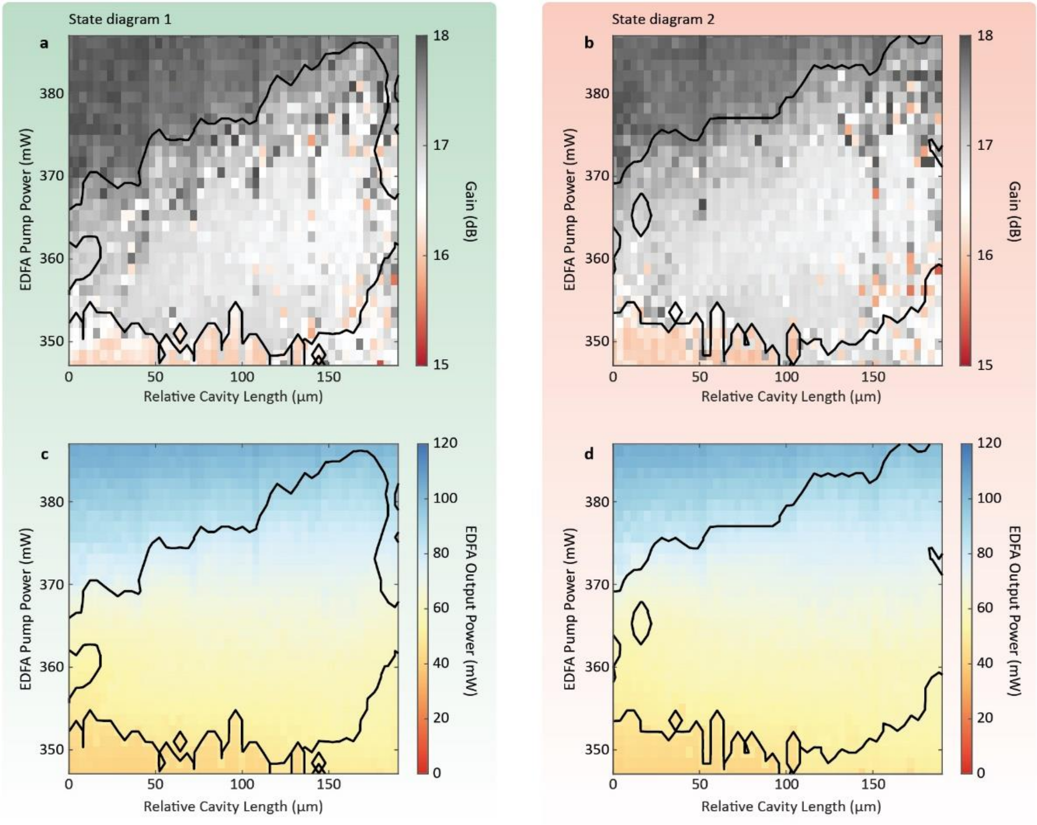

Figure S5 Additional measurements for the 'State Diagram 1 and 2' presented in Fig. S4a and c, respectively. **a** Optical gain of the EDFA for the 'State Diagram 1'. **b** Same as panel b, for the 'State Diagram 2'. **c** Intracavity power measured at the output of the amplifier or, equivalently, at the input of the microcavity, for the 'State Diagram 1'. **d** Same as panel c, for the 'State Diagram 2'.

The frequency f of a selected mode of the microresonator (around 1543 nm) shown in Fig. S6a,b is extracted from the microcavity resonances detected via laser scanning spectroscopy. Figure 4c of the main text depicts a typical example where the shift of the microcavity resonance is clearly visible. Since the shift of the microcavity resonance dominates the variation of this frequency, it allows evaluating the change of the microcavity refractive index n_a under operating conditions. We can use the general resonance frequency formula $f = M\ c\ L_a^{-1}\ n_a^{-1}$, where M is an integer, L_a is the microcavity length, and c is the speed of light. This formula allows one to obtain a rough monitoring of the refractive index change δn_a for the microcavity. Assuming that the dominant change is via the refractive index and not the length, such a change is proportional to the variation of the frequency $-\delta f\ f^{-1} = \delta n_a n_a^{-1}$. For the range in Fig. S6a,b ($\delta f = [0, 6]$ GHz) this corresponds to a relative refractive index variation in the range of $|\delta n_a n_a^{-1}| = 2 \times 10^{-5}$ across the soliton region. Such a change is induced by the variation of the power in the comb light, as evidenced in Fig. S5c,d that depict the optical comb power coupled to the microring. Across the soliton region, the power variation is about 10 mW. Thermal effects in the microcavity dominate such a refractive index change, which increases proportionally to the optical power.

To calculate the average detuning shown in Fig. S6c,d, we extract the detuning distributions for all the valid points on the map (similar to those in Fig. S3g,h) and take the average value. Fig. S6c reports, for convenience, the same data as Fig. 4f in the main text. These measurements confirm the clear locking of the soliton and CW states on the red-detuned and blue-detuned slopes that we discussed in the main text. Such a locking occurs despite the strong thermal detuning of the microcavity resonances, as we also commented in the main paper, due to the presence of another nonlinear phenomenon in the amplifier. Evidence of such a nonlinearity can be found in the variation of the free-spectral range of the soliton states that we discuss in the analysis of Fig. S6e,f and Fig. S7.

We extracted the free-spectral range of the soliton states shown in Fig. S6e,f by fitting the frequency position of the soliton modes versus the mode numbers, as also done in Refs.[1,3]. Here, we consider the repetition rate of a single soliton as ≈ 48.9 GHz. The change of free-spectral range under operating conditions spans about 2 MHz, which accounts for the group velocity mismatch that the soliton solutions admit[1–3].

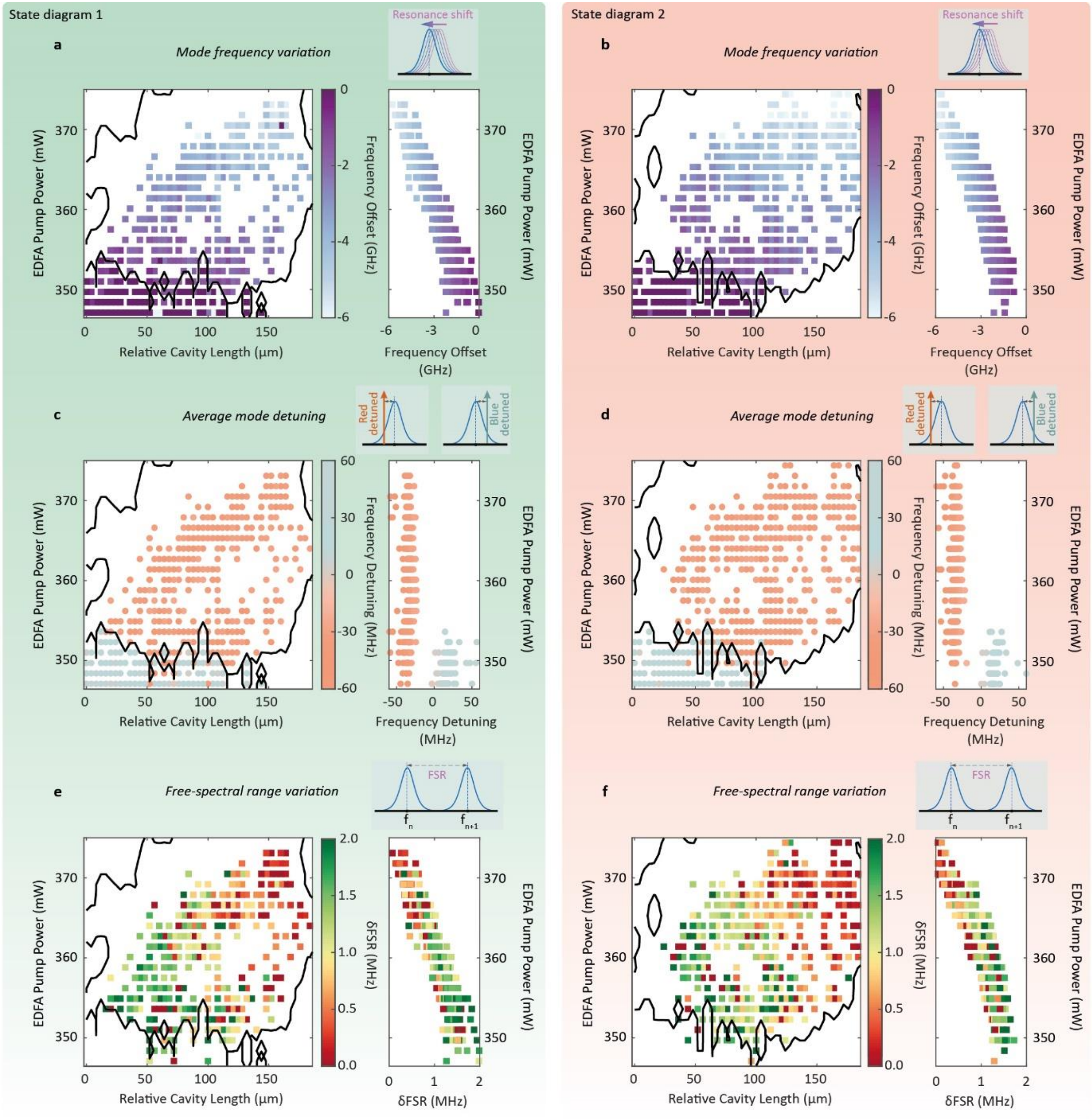

Figure S6 Analysis of the frequency positions of the stable states. Data extracted from the laser scanning spectroscopy. Black lines mark soliton and CW region boundaries. **a** Variation of the frequency position of a selected resonance of the microcavity (here, we choose the mode centred approximately at 1543 nm). The colour code in the map follows the inset (right), showing the values of the frequency shift for the microcavity resonance. **b** Same as panel a, now for the 'State Diagram 2'. **c** Variation of the average detunings, calculated as the mean frequency difference between each oscillating microcomb laser line and the corresponding microcavity resonance centre across all microcomb lines. The colour code in the map follows the colours of the inset (right), where the values of the detunings versus EDFA pump power are shown. **d** Same as panel c, but for the 'State Diagram 2'. **e** Free-spectral range (FSR) variation associated with the soliton states (FSR~48.9 GHz) obtained across the mapped values. The colour code in the map follows the inset (right) showing the free-spectral range variation values. **f** Same as panel e, but for the 'State Diagram 2'.

The free-spectral range variation allows us to evaluate the refractive index change in the amplifying cavity indirectly. The position of the oscillating lines dominates such a variation. Hence, we can assume that the relative deviation of the free-spectral range of the soliton solutions

$\delta F\ F^{-1}$, is equal to the relative variation of the amplifier cavity free-spectral range, $F_b \approx 77$ MHz, with $F_b = c\ L_b^{-1}\ n_b^{-1}$. The variation of F_b under operating conditions is dependent on the changes in the amplifier cavity length L_b and refractive index n_b with $-\delta F\ F^{-1} = -\delta F_b\ F_b^{-1} = \delta n_b n_b^{-1} + \delta L_b L_b^{-1}$. From this relationship, we can extract the relative refractive index change $\delta n_b n_b^{-1}$, because the variation δL_b is known, it is indeed the relative cavity length variation that we are reporting in the abscissa of all the maps. Figures S7a,b show such relative refractive index change $\delta n_b n_b^{-1}$ that we have extracted from the free-spectral range variation maps in Fig. S6e,f. Here, we observe a variation in the order of $|\delta n_b n_b^{-1}| \approx 1 \times 10^{-4}$.

The most important result of Fig. S7a,b is that the refractive index increases with the EDFA pump power, as we observed for the microcavity in Fig. S6a,b. This indicates that the amplifier has a dominating focusing thermal nonlinearity***, in accordance with the literature of EDFAs[24]. The variation of refractive index within the amplifier is $|\delta n_b n_b^{-1}| \approx 1 \times 10^{-4}$ is larger than the variation in the microcavity ($|\delta n_a n_a^{-1}| = 2 \times 10^{-5}$). In the amplifier, however, such a change is induced by both the EDFA pump variation (of about 30 mW) and the amplified signal variation (10 mW). Given that the power variations of these two fields are within the same range, it is reasonable to assume that the comb contribution to this change is at least in the order of 10^{-5}, comparable with the microcavity refractive index change. When the intracavity optical comb power varies, hence, the amplifier and the microring experience a refractive index change of the same sign and of a similar amount. We recall that, by definition, a nonlinear coefficient measures the refractive index variation as a function of the optical power. These measurements, hence, show that in the soliton regime the slow nonlinearities of the amplifier and the microcavity have the same sign. The results also suggest that these nonlinearities are in the same order of magnitude.

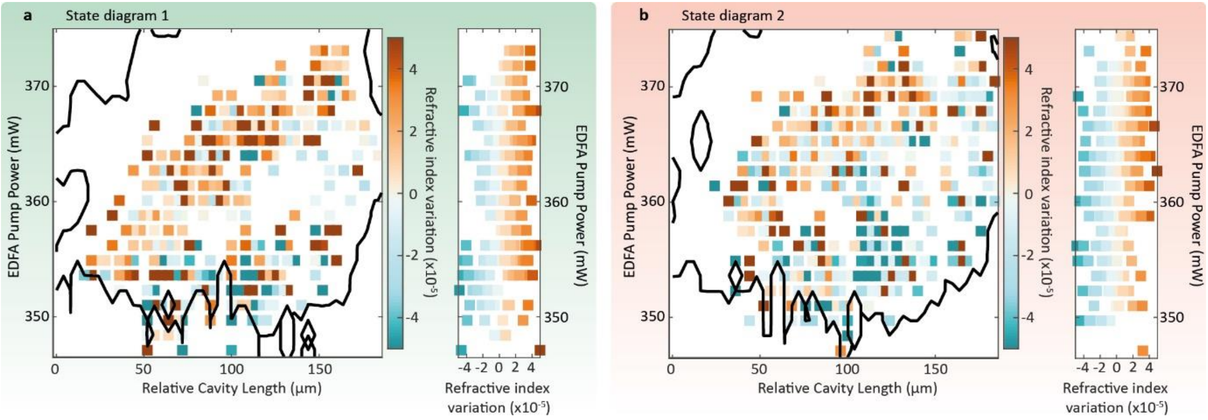

Figure S7 Analysis of the relative refractive index variation in the amplifier cavity. Here we show the relative refractive index variation of the amplifier under operating condition calculated as $\delta n_b n_b^{-1} = -\delta F\ F^{-1} - \delta L_b L_b^{-1}$. **a** Relative change of the refractive index for the soliton states obtained across the mapped values, extracted from the free-spectral range variation of the 'State Diagram 1'. The colour code in the map follows the inset showing the refractive index change. **b** Same as panel a for the 'State Diagram 2'.

*** The other relevant physical phenomenon is the gain-induced change of refractive index. This decreases the refractive index for increasing gain and, accordingly, increasing EDFA pump power. For this reason, this cannot be the dominating effect in these measurements.

It is important to stress that, in terms of nonlinearity, the gain dependency still results in a focusing nonlinearity for the comb field, because the gain saturates (and hence decreases, effectively leading to a larger refractive index) for increasing comb powers.

In our modelling, we take this into account with the term $\Theta\ g_P$ in the detuning. Indeed, we use a negative value for Θ which results in a decrease of the refractive index for increasing gain g_P. This terms results in a focusing nonlinearity when the gain g saturates with the field b.

S2.2 Experimental diagram of states at 14 dB losses and system dynamic response

In the previous section, we showed that the experimental state diagram remained almost unchanged for fixed intracavity losses. Soliton and CW states are repeatable, i.e., they appear in independent runs for the same EDFA pump power and laser cavity length settings, with very similar refractive index variations (within the accuracy of our estimation), and minor differences in the intracavity energies.

We now discuss the system behaviour when the intracavity losses are varied. Blue-detuned and red-detuned types of attractors still appear; however, this generally occurs for different pump power values and relative cavity lengths. In addition, by varying the losses, the intracavity saturation energy is also affected: this typically allows reaching different nonlinear regimes.

Figure S8 shows the resulting state diagram obtained for an intracavity loss (14 dB) lower than the one considered in the previous section (16 dB): blue-detuned states take the form of Turing patterns. The output drop power for these states is about 15 mW. At these powers, the microcavity Kerr nonlinearity is strong enough to induce modulational instability; hence the CW states naturally evolve into patterns. At higher EDFA pump powers, we observe red-detuned soliton states again. Here two and three soliton states appear, with output drop powers of about 20 mW. The global parameters (in this case, losses) can control the number of solitons by operating the system at different intracavity energies.

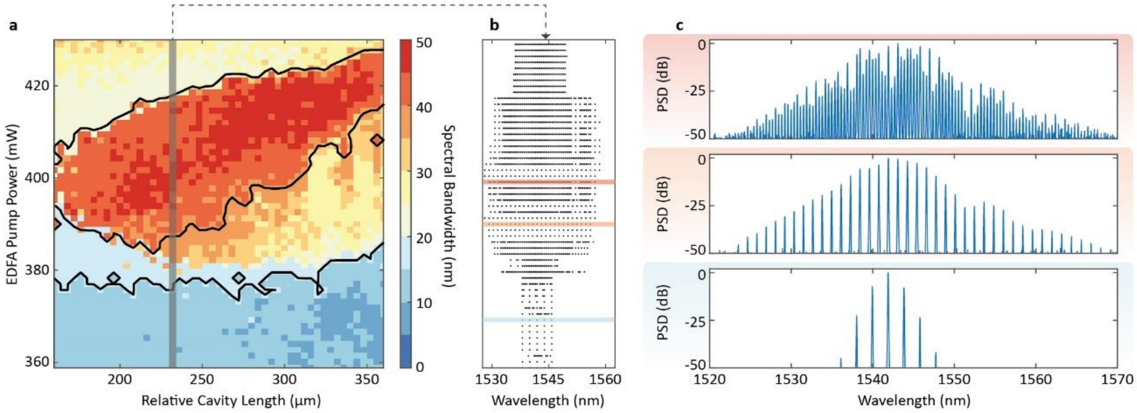

Figure S8. Experimental state diagram for a microresonator-filtered fibre laser with intracavity losses set to 14 dB. The delay axis uses the same zero definition as Fig. S4. **a** Spectral bandwidth (calculated as the bandwidth at -40dB from the maximum), in colour code, of the laser state as a function of the cavity length delay and EDFA pump power. Soliton states are found in the red region with the broadest bandwidths, while Turing patterns appear at the lowest EDFA values (in blue). The lasing regime is unstable outside these regions, which are roughly defined by the black border lines. During the experimental acquisition, the temperature was maintained at 40 °C within a fluctuation of a few degrees. **b** Optical spectra of the states at the delay 240 μm, identified by the vertical grey line in panel a. The black dots represent the frequency position relative to the maximum value of each comb mode. **c** From top to bottom, power spectral densities (PSDs) of the optical spectra for EDFA powers of 398 mW, 391 mW and 373 mW, respectively, as indicated by the corresponding coloured highlights in panel b.

Across all maps, we consistently observe a clear transition between blue-detuned and red-detuned dominant attractor states determined by the EDFA pump power level. For appropriate values of losses and main cavity length, the EDFA pump power appears to control an equilibrium point between the two types of states. Moreover, the analysis of the previous section shows that the refractive indices of the two cavities vary within a similar range across the maps. We interpret this as evidence of a similar order of magnitude between the slow, time-dependent nonlinearities of the two cavities.

The change in their equilibrium induces in the system two different families of attractors. These two families are characterised by microcomb lines locked on the blue-detuned and red-detuned sides of the microcavity resonances, respectively. Attractors with microcomb lines locked on the

blue-detuned side of the microcavity resonances (blue-shifted detunings as shown in Fig. S3e-h and Fig. S6c,d, blue values) result in CW states or Turing states. Such blue-detuned states appear when the dominating nonlinear effect takes place in the microcavity. Across our maps, we observe this behaviour at lower EDFA powers.

On the other hand, attractors with microcomb lines locked on the red-detuned side of the microcavity resonances (red-shifted detunings as shown in Fig. S3a-d,g-h and Fig. S6c,d, red values) lead to soliton states. Such red-detuned states appear when the dominating nonlinear effect takes place in the amplifier cavity. Across our maps, we observe this behaviour at high EDFA powers.

In agreement with the literature on EDFAs[24], increasing the EDFA pump power also raises the nonlinearity in the amplifier. This phenomenon, also according to the physics described in our modelling in Section S1, is induced by the nonlinear gain response of the EDFA. Typically, the latter provides a small variation in the system focusing nonlinearity, primarily dominated by the large focusing thermal effect[24] observed in Fig. S7. To identify the change of balance between the two nonlinearities, we use, then, a different approach. Since the two slow nonlinearities of the microring and amplifying cavity have different physical natures and timescales, we verify our hypothesis on the operating regimes by studying the recovery time of the attractors. For blue-detuned Turing states, we expect a recovery dynamic dominated by the thermal nonlinearity of the microcavity. For red-detuned soliton states, conversely, we expect a recovery time compatible with the gain nonlinearity of the amplifier.

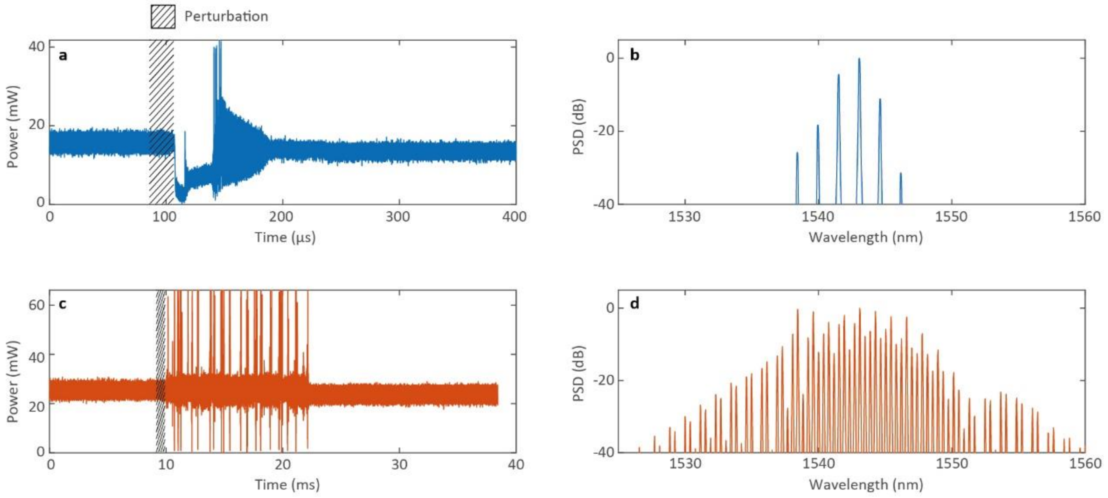

Figure S9 Perturbation recovery. The stationary state is perturbed with a steep variation in the voltage driving the EDFA pump power. **a** Recovery of a Turing pattern state. The power associated with the state is shown in blue, while the black shaded region indicates the time at which the EDFA pump power is modified to perturb the state. The system recovers to a state with similar average power and spectral shape after 0.1ms, which is in the temporal scale of the microcavity thermal nonlinearity response. **b** Corresponding optical spectrum of the recovered state in a. **c** Same as panel a, for a multi-soliton state. Note that the recovery time is on the order of 10 ms, dictated by the Erbium gain. **d** Corresponding optical spectrum for the recovered state in panel c.

Figure S9 shows the laser output while abruptly changing the EDFA pump power for a Turing pattern and a soliton state. Here we explicitly show the transitory state that we observe when we abruptly decrease the EDFA pump power (of about 8 mW for the two cases). In both cases, the microcomb regains a state with very similar physical properties, despite the strong variation in output power following the perturbation. For a low EDFA pump power leading to Turing states (Fig. S9a), the system recovers on a timescale of 100 μs which is governed by thermal changes in the microcavity. In contrast, for soliton states (Fig. S9b), the system recovers in a timescale of 10 ms - the Erbium-doped fibre gain response time. These measurements confirm that the system is dominated by two different nonlinearities when it converges to the two different attractors. The microcavity thermal nonlinearity dominates at low pump powers and induces blue-detuned

states. In contrast, the amplifier nonlinearity, which increases with the EDFA pump power, prevails in the high-energy soliton region.

S3. Numerical analysis of the dynamic system response.

In this section, we show that the formation of blue-detuned patterns and red-detuned solitons are respectively dominated by the time constants of the microcavity thermal nonlinearity and of the amplifier gain nonlinearity also in our theory.

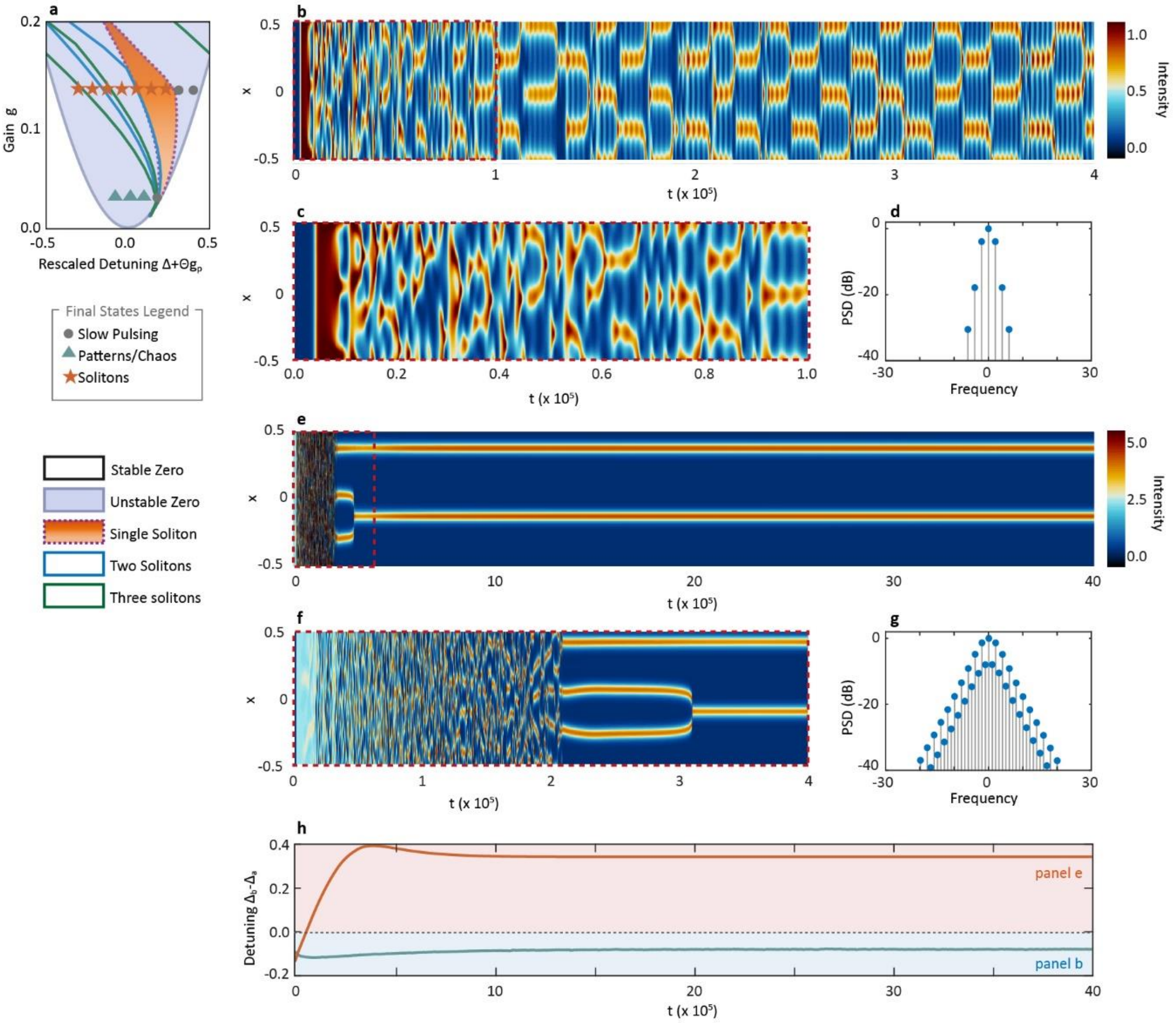

Figure S10 Start-up in the presence of gain-induced nonlinearity and thermal nonlinearity. a Stability region and a selection of steady states for the system. The colour of each marker represents the type of steady-state solution reached by the system after start-up from the noise for different values of the rescaled detuning $\Delta + \Theta g_P$ and gain g. **b** Pseudo-colour map of the electric field intensity in the microcavity as a function of the position in the microresonator x (normalised against the microcavity roundtrips) and time t (normalised against main-cavity roundtrips). The system parameters are $\Gamma_T = 2, \tau_T = 8 \times 10^3, \eta = 0.1, \tau_g = 4 \times 10^4, \Theta = -15, g = 0.03, \Delta + \Theta g_p = -0.0909$. **c** Enlarged view of panel b for the first 10^4 main-cavity roundtrips, highlighting the start-up of the pattern-like solution within a timescale compatible with the thermal time constant $\tau_T = 8 \times 10^3$. **d** Output power spectral density (PSD) of the a field. **e-g** Same as panels b,c and d, but for parameters $\Gamma_T = 2, \tau_T = 8 \times 10^3, \eta = 0.1, \tau_g = 4 \times 10^4, \Theta = -15, g = 0.135, \Delta + \Theta g_p = -0.1342$. Panel f illustrates the start-up of the two-soliton state from noise within a time frame compatible with the slower timescale $\tau_g = 4 \times 10^4$. **h** Temporal evolution of the effective detuning $\Delta_b - \Delta_a$ for the low-gain pattern-like states (blue line) and the two-soliton states (orange line).

In our theoretical modelling, Eqs. (1-4), the thermal refractive index of the microcavity is accounted for in Eq. (4). The laser refractive index dependence on the pump power, conversely, is described by the term $\Theta\, g$ in the equation for Δ_b, with the gain term given by Eq. (3). As the gain saturates, this term induces a red-detuned nonlinearity controlled by the pumping term g_P, modelling the EDFA pump, and in turn, the gain saturation induced nonlinearity – see, e.g., Ref.[24]. Using the same parameters as in Section S1 but with constant $\kappa = 2\pi, \Gamma_T = 2, \eta = 0.1, \Theta = -15$, we

numerically investigated the dynamic evolution of the complete system of Eqs. (1-4) for different initial detuning Δ and with two different time constants. As discussed in Section S1, we set the thermal decay time of the microcavity to $\tau_T = 8 \times 10^3$ and the saturation time of the gain to $\tau_g = 4 \times 10^4$.

We start by calculating the start-up region in terms of the global parameters g_P and the initial detuning Δ in Fig. S10a. Figure S10a summarises the results for this series of simulations. Here we use a series of coloured markers denoting the type of final steady-state (grey dots - slow pulsing, blue triangles – pattern, orange stars - soliton) for different initial detuning and gain values.

As it can be seen from this graph, at low pump gain (e.g., $g_P = 0.03$) the start-up region does not admit soliton states. Here, we observed the emergence of pattern-like (blue triangle markers), blue-detuned types of states due to the presence of a focusing microcavity thermal nonlinearity induced by the term $\Gamma_T = 2$ in Eq. (4). These types of states are the dominant attractors. In Fig. S10b-d, we illustrate the formation and evolution of a typical pattern-like solution (panels S10b,c, pseudo-colour plot of the temporal evolution of the microcavity field a; panel S10d, output spectrum).

At higher gain (g_P=0.135), the system dramatically changes its dynamics. Looking at the map in Fig. S10a, we see that the start-up region admits soliton states. We verified by numerical propagation (orange stars) that they appeared for $\Delta + \Theta\ g_P \in$ [-0.3132, 0.2237]. We show the formation of a typical two-soliton state for $\Delta + \Theta\ g_P$ =0.1342 in Fig. S10e-g.

Figure S10h includes the evolution of the effective detuning $\Delta_b - \Delta_a$ for both the low (g_P=0.03, blue line) and high (g_P=0.135, red line) gain cases. At lower gains, where the dynamics converge to a pattern-like solution (blue line), the focusing thermal nonlinearity squeezes the effective detuning values $\Delta_b - \Delta_a$ into a smaller value. In the high-gain case (orange line), conversely, the effective detuning $\Delta_b - \Delta_a$ moves initially towards the blue, but then it settles for a sharp red-shift, enabling the soliton state formation.

Our numerical simulations also well reproduce the experimentally observed temporal dynamics. The low gain dynamics of Fig. S10b-d, in particular, compare well with the observation of a pattern state formation in Fig. S9a. The experimental and numerical spectra show very good qualitative agreement. Most importantly, our experiments suggest a recovery of the pattern on the thermal timescale of the microcavity τ_T. This is consistent with our simulations, where we found that the main pattern forms indeed in the first $\tau_T = 8 \times 10^3$ main-cavity roundtrips. In this specific example, the pattern continues to evolve slowly. As discussed in Ref.[3], our system produces stable patterns by including the group velocity mismatch, which we have constrained to be zero for simplicity. Experimentally, we do not expect this restriction to happen in the region where we measure the patterns.

More significantly, the results depicted in Fig. S10e,f are in excellent agreement with the experimental recovery of the multi-soliton state displayed in Fig. S9c. This shows a long timescale dynamic, clearly dominated by the gain recovery time constant $\tau_g = 4 \times 10^4$, in close analogy with Fig. S9c, which is also dominated by the Erbium time constant.

Finally, we note that the model moves from a pattern to a soliton dynamic when incrementing the gain by about 1 dB, which roughly provides the gain change necessary to move from the pattern region to the soliton region, as visible from Fig. S10a. This value is compatible with the experimental gain variation we observed across the maps (see Fig. S5a,b) between the blue-detuned and red-detuned regions.

References

1. Bao, H. *et al.* Laser cavity-soliton microcombs. *Nat. Photonics* **13**, 384–389 (2019).
2. Cutrona, A. *et al.* Temporal cavity solitons in a laser-based microcomb: a path to a self-starting pulsed laser without saturable absorption. *Opt. Express* **29**, 6629–6646 (2021).
3. Bao, H. *et al.* Turing patterns in a fiber laser with a nested microresonator: Robust and controllable microcomb generation. *Phys. Rev. Res.* **2**, 023395 (2020).
4. Scroggie, A. J., Firth, W. J. & Oppo, G.-L. Cavity-soliton laser with frequency-selective feedback. *Phys. Rev. A* **80**, 013829 (2009).
5. Paulau, P. V., Gomila, D., Colet, P., Malomed, B. A. & Firth, W. J. From one- to two-dimensional solitons in the Ginzburg-Landau model of lasers with frequency-selective feedback. *Phys. Rev. E* **84**, 036213 (2011).
6. Atai, J. & Malomed, B. A. Stability and interactions of solitons in two-component active systems. *Phys. Rev. E* **54**, 4371–4374 (1996).
7. Malomed, B. A. Solitary pulses in linearly coupled Ginzburg-Landau equations. *Chaos Interdiscip. J. Nonlinear Sci.* **17**, 037117 (2007).
8. Atai, J. & Malomed, B. A. Bound states of solitary pulses in linearly coupled Ginzburg-Landau equations. *Phys. Lett. A* **244**, 551–556 (1998).
9. Matera, F., Romagnoli, M., Settembre, M., Tamburrini, M. & Peroni, M. Evaluation of chromatic dispersion in erbium doped fibre amplifiers. *Electron. Lett.* **27**, 1867 (1991).
10. Tartwijk, G. H. M. van & Agrawal, G. P. Maxwell–Bloch dynamics and modulation instabilities in fiber lasers and amplifiers. *JOSA B* **14**, 2618–2627 (1997).
11. Van Tartwijk, G. H. & Agrawal, G. P. Laser instabilities: a modern perspective. *Prog. Quantum Electron.* **22**, 43–122 (1998).
12. Spinelli, L., Tissoni, G., Lugiato, L. A. & Brambilla, M. Thermal effects and transverse structures in semiconductor microcavities with population inversion. *Phys. Rev. A* **66**, 023817 (2002).
13. Scroggie, A. J., McSloy, J. M. & Firth, W. J. Self-propelled cavity solitons in semiconductor microcavities. *Phys. Rev. E* **66**, 036607 (2002).
14. Pasquazi, A. *et al.* Micro-combs: A novel generation of optical sources. *Phys. Rep.* **729**, 1–81 (2018).
15. Rowley, M. *et al.* Thermo-optical pulsing in a microresonator filtered fiber-laser: a route towards all-optical control and synchronization. *Opt. Express* **27**, 19242–19253 (2019).
16. Carmon, T., Yang, L. & Vahala, K. J. Dynamical thermal behavior and thermal selfstability of microcavities. *Opt. Express* **12**, 4742–4750 (2004).
17. Grudinin, I., Lee, H., Chen, T. & Vahala, K. Compensation of thermal nonlinearity effect in optical resonators. *Opt. Express* **19**, 7365 (2011).
18. Savchenkov, A. A., Matsko, A. B. & Maleki, L. On Frequency Combs in Monolithic Resonators. *Nanophotonics* **5**, 363–391 (2016).
19. Voloshin, A. S. Dynamics of soliton self-injection locking in optical microresonators. *Nat. Commun.* **12**, (2021).
20. Kondratiev, N. M. & Lobanov, V. E. Modulational instability and frequency combs in whispering-gallery-mode microresonators with backscattering. *Phys. Rev. A* **101**, 013816 (2020).
21. Bao, H. *et al.* Type-II micro-comb generation in a filter-driven four wave mixing laser [Invited]. *Photonics Res.* **6**, B67–B73 (2018).
22. Kippenberg, T. J., Gaeta, A. L., Lipson, M. & Gorodetsky, M. L. Dissipative Kerr solitons in optical microresonators. *Science* **361**, eaan8083 (2018).
23. Pasquazi, A. *et al.* Micro-combs: A novel generation of optical sources. *Phys. Rep.* **729**, 1–81 (2018).
24. Barmenkov, Yu. O., Kir'yanov, A. V. & Andrés, M. V. Resonant and thermal changes of refractive index in a heavily doped erbium fiber pumped at wavelength 980nm. *Appl. Phys. Lett.* **85**, 2466–2468 (2004).
